# *Prototheca wickerhamii* Cutaneous and Systemic Infections

**DOI:** 10.4269/ajtmh.14-0082

**Published:** 2014-10-01

**Authors:** Deng-Wei Chou, Kuo-Mou Chung, Chao-Tai Lee

**Affiliations:** Department of Internal Medicine, Tainan Municipal Hospital, Tainan City, Taiwan; Department of Clinical Laboratory, Tainan Municipal Hospital, Tainan City, Taiwan

## Abstract

*Prototheca wickerhamii,* an environmental alga, rarely causes human infections. We present a case of *Prototheca wickerhamii* cutaneous and systemic infections in an 85-year-old male with adrenal insufficiency. This organism was identified by morphological features and microbiological tests. The patient was successfully treated with ketoconazole.

An 85-year-old male presented with a fever lasting for 2 days. He had a history of adrenal insufficiency with prednisolone use for 2 years. Pruritic erythematous maculopapules on his lower extremities appeared 1 year ago. On examination, he was febrile, tachycardic, and tachypneic. Multiple erythematous plaques were accompanied by papules, shallow ulcers, and crusts on his four limbs (Figure 1). Empirical piperacillin/tazobactam therapy was initiated. Blood cultures obtained on admission were positive after 3 days of incubation. Gram stain revealed spherical Gram-positive organisms of various sizes (Figure 2). A subculture on a blood agar plate showed milky white yeast-like colonies (Figure 3). A lactophenol cotton blue wet mount preparation disclosed characteristic endosporulating sporangia (Figure 4). The organisms isolated from both blood and cutaneous wound cultures were identified as *Prototheca wickerhamii* using the API 20C identification system (bioMérieux, Marcy l’Etoile, France). Ketoconazole therapy was started on hospital Day 6. His clinical condition and cutaneous lesions improved with ketoconazole for a total of 4 weeks.

**Figure 1.**
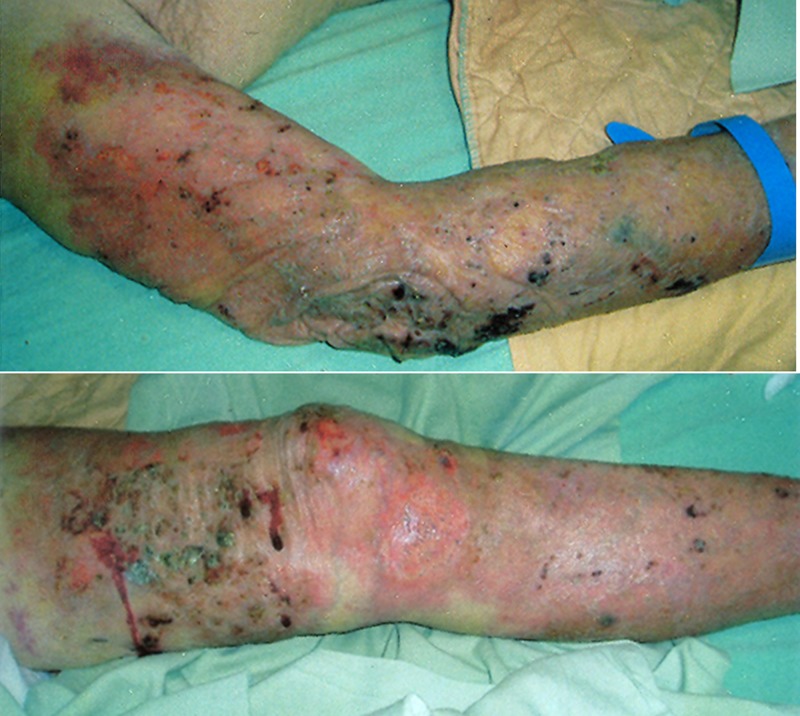
Multiple erythematous plaques are accompanied by papules, shallow ulcers, and crusts on his right upper limb and left lower limb.

**Figure 2.**
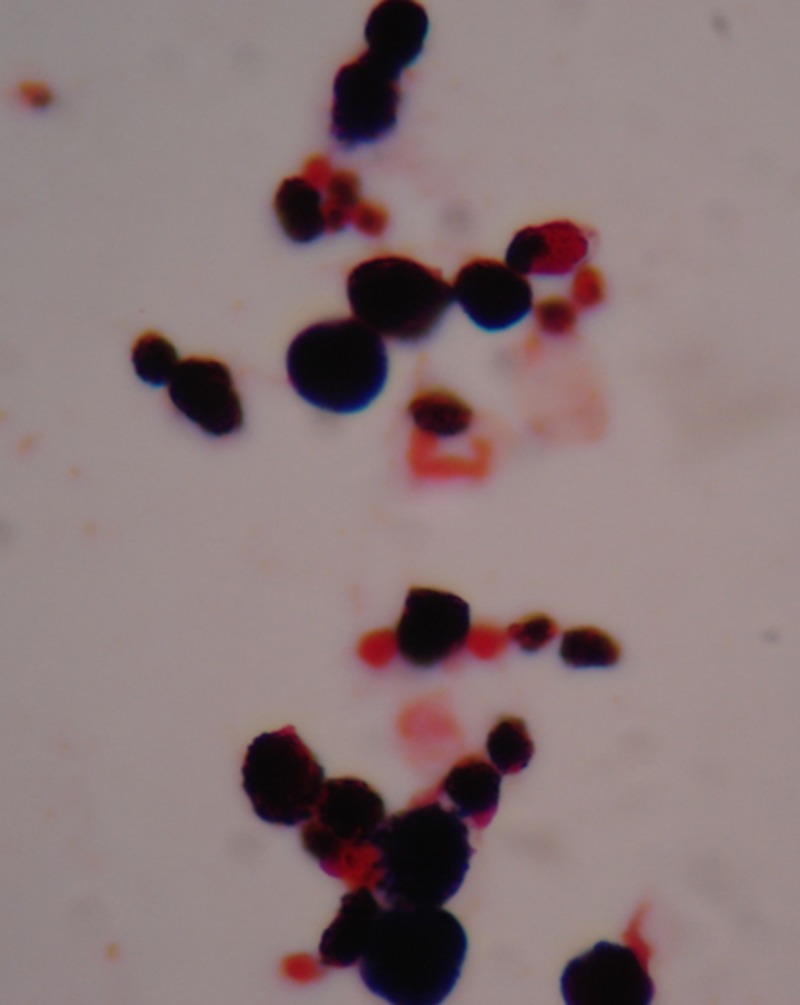
Gram stain of blood culture reveals spherical Gram-positive organisms of various sizes resembling yeast. Magnification, ×1,000.

**Figure 3.**
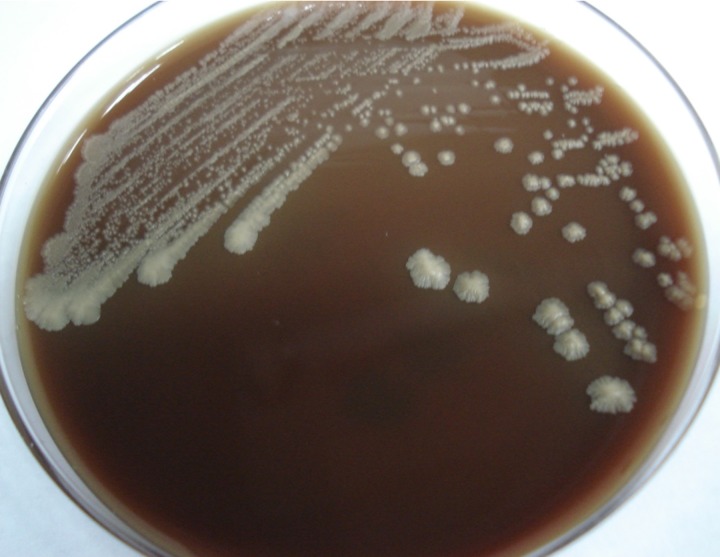
Milky white yeast-like colonies are observed on blood agar plate after incubation at 35°C for 3 days.

**Figure 4.**
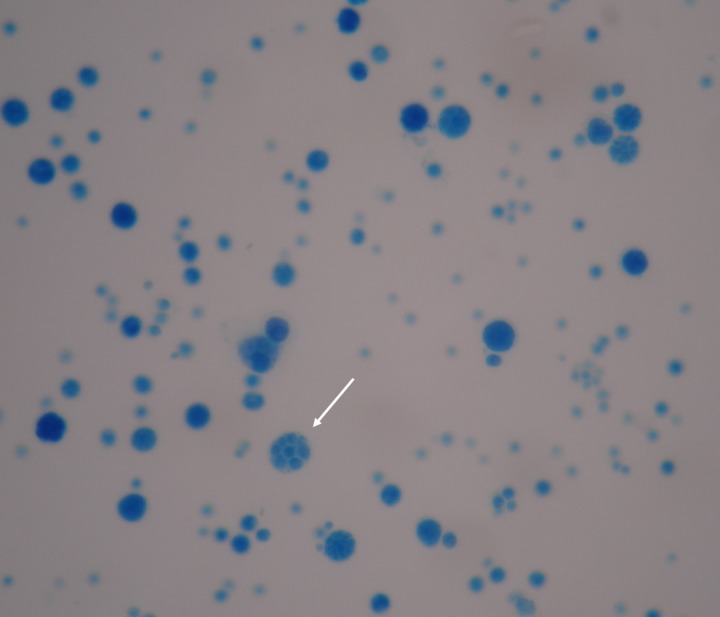
Wet-mount preparation with lactophenol cotton blue discloses spherical sporangia containing multiple endospores with symmetrical arrangement. Magnification, ×1,000.

*Prototheca wickerhamii* is an achlorophyllic alga and is ubiquitous in nature, which can cause human infections. The definite diagnosis usually depends on morphological identification of the organisms in wet slide preparations of cultures and/or direct identification in tissue specimens.^1^
